# Parallel Analysis of Cystic Fibrosis Sputum and Saliva Reveals Overlapping Communities and an Opportunity for Sample Decontamination

**DOI:** 10.1128/mSystems.00296-20

**Published:** 2020-07-07

**Authors:** Junnan Lu, Lisa A. Carmody, Kristopher Opron, Richard H. Simon, Linda M. Kalikin, Lindsay J. Caverly, John J. LiPuma

**Affiliations:** a Department of Pediatrics, University of Michigan Medical School, Ann Arbor, Michigan, USA; b Department of Internal Medicine, University of Michigan Medical School, Ann Arbor, Michigan, USA; UCSF

**Keywords:** microbiome, airway infection, microbial community dynamics

## Abstract

Culture-independent studies of the cystic fibrosis (CF) airway microbiome typically rely on expectorated sputum to assess the microbial makeup of lower airways. These studies have revealed rich bacterial communities. There is often considerable overlap between taxa observed in sputum and those observed in saliva, raising questions about the reliability of expectorated sputum as a sample representing lower airway microbiota. These concerns prompted us to compare pairs of sputum and saliva samples from 10 persons with CF. Using 16S rRNA gene sequencing and droplet digital PCR (ddPCR), we analyzed 37 pairs of sputum and saliva samples, each collected from the same person on the same day. We developed an *in silico* postsequencing decontamination procedure to remove from sputum the fraction of DNA reads estimated to have been contributed by saliva during expectoration. We demonstrate that while there was often sizeable overlap in community membership between sample types, expectorated sputum typically contains a higher bacterial load and a less diverse community compared to saliva. The differences in diversity between sputum and saliva were more pronounced in advanced disease stage, owing to increased relative abundance of the dominant taxa in sputum. Our effort to model saliva contamination of sputum *in silico* revealed generally minor effects on community structure after removal of contaminating reads. Despite considerable overlap in taxa observed between expectorated sputum and saliva samples, the impact of saliva contamination on measures of lower airway bacterial community composition in CF using expectorated sputum appears to be minimal.

**IMPORTANCE** Cystic fibrosis is an inherited disease characterized by chronic respiratory tract infection and progressive lung disease. Studies of cystic fibrosis lung microbiology often rely on expectorated sputum to reflect the microbiota present in the lower airways. Passage of sputum through the oropharynx during collection, however, contributes microbes present in saliva to the sample, which could confound interpretation of results. Using culture-independent DNA sequencing-based analyses, we characterized the bacterial communities in pairs of expectorated sputum and saliva samples to generate a model for “decontaminating” sputum *in silico*. Our results demonstrate that salivary contamination of expectorated sputum does not have a large effect on most sputum samples and that observations of high bacterial diversity likely accurately reflect taxa present in cystic fibrosis lower airways.

## Abstract (precis)

Cystic fibrosis is an inherited disease characterized by chronic respiratory tract infection and progressive lung disease. Studies of cystic fibrosis lung microbiology often rely on expectorated sputum to reflect the microbiota present in the lower airways. Passage of sputum through the oropharynx during collection, however, contributes microbes present in saliva to the sample, which could confound interpretation of results. Using culture-independent DNA sequencing-based analyses, we characterized the bacterial communities in pairs of expectorated sputum and saliva samples to generate a model for “decontaminating” sputum *in silico*. Our results demonstrate that salivary contamination of expectorated sputum does not have a large effect on most sputum samples and that observations of high bacterial diversity likely accurately reflect taxa present in cystic fibrosis lower airways.

## INTRODUCTION

Despite the considerable advances made in the care of persons with cystic fibrosis (CF) and the resultant increase in life expectancy in this population during the last 3 decades, chronic airway infection leading to respiratory failure remains the primary cause of death. Initially, studies of CF airway infection focused on human-pathogenic bacterial species (*Staphylococcus* and *Haemophilus*) recovered in cultures of respiratory secretions. The bacteria of interest in CF gradually expanded to include several opportunistic pathogens including *Pseudomonas*, *Burkholderia*, *Achromobacter*, *Stenotrophomonas*, and mycobacteria (1). The advent of culture-independent approaches to study the human microbiome has, more recently, enabled a deeper exploration of CF airway microbiology, and studies employing these methods have indicated that CF airways likely harbor more diverse bacterial communities than previously appreciated (2, 3). These new insights hold promise to translate into novel approaches to better manage airway infection, further reducing respiratory system-associated morbidity and mortality in CF.

Sampling of the lower airways to assess the microbial communities therein presents a challenge, however. Although bronchoscopy has been used to obtain airway secretions (i.e., via bronchoalveolar lavage [BAL]) in several studies of CF microbiology (4–10), this approach is invasive, not without risk, and not feasible for studies requiring analysis of serial samples from individuals across a large subject cohort. Expectorated sputum, therefore, has been most often used to address the dynamics of airway bacterial communities in CF. Because this respiratory tract specimen passes through the nonsterile upper airway and oropharynx, however, it must be assumed to be “contaminated” with microbes residing in these sites, raising concerns about the reliability of sputum in reflecting lower airway microbial communities. The degree to which upper airway microbes contribute to the bacterial communities measured in expectorated sputum—and, by extension, the degree to which these species can be held to account for the conclusion that CF lower airways harbor rich bacterial communities—is difficult to ascertain.

To improve our understanding of the relationship between upper and lower airway bacterial communities, we analyzed pairs of expectorated sputum and saliva samples collected from individuals with CF. We estimated the effect of saliva contamination on expectorated sputum and propose a model to account for the contribution of salivary microbiota to measures of sputum bacterial community composition.

## RESULTS

### Subjects, samples, and DNA sequencing controls.

Ten adult subjects with CF provided 37 same-day pairs of sputum and saliva samples. Subject demographics and sample characteristics are summarized in Table 1. The median subject age was 32 years. The median percent predicted forced expiratory volume in 1 s (ppFEV_1_) was 61, measured within 1 to 45 days of the collection day. The number of sample pairs per subject ranged from one to five. For subjects with more than one sample pair, the period of time between the first and last sample pair averaged 94 days (range 8 to 330 days), and the time between consecutive samples averaged 32 days (range 7 to 253 days). Six sample pairs were collected during antibiotic treatment (i.e., treatment other than maintenance antibiotic use). Only one subject had advanced lung disease, but all spontaneously expectorated sputum on a daily basis.

**TABLE 1:** Subject and sample characteristics for 37 pairs of expectorated sputum and saliva

Subject	No. of pairs	Sex	CFTR genotype	Age (yr) at 1st sample	Disease stage^*a*^
1	5	F	F508del homozygous	19	Int
2	5	F	F508del homozygous	30	Early/Int
3	5	F	F508del heterozygous	26	Int/Adv
4	5	M	F508del heterozygous	21	Early
5	5	F	F508del homozygous	56	Early/Int
6	1	F	F508del heterozygous	51	Int
7	2	M	F508del heterozygous	32	Int
8	3	F	F508del homozygous	29	Int
9	3	M	F508del heterozygous	35	Adv
10	3	F	Other	32	Int

a Disease stage was assigned as described in the text. Int, intermediate; Adv, advanced.

The median DNA sequencing error rate, based on mock community analyses, was 0.032% (range 0.025% to 0.036%). The median bacterial load of reagent controls was 4.2 × 10^3^ (range 3.8 × 10^3^ to 4.5 × 10^3^) 16S rRNA gene copies/ml, which was more than 5 logs lower than the median bacterial load of sputum and saliva samples. Neither the reagent nor water controls amplified during library construction. The mean pairwise theta-YC similarity of 59 generous donor control replicates was 0.983 (range 0.931 to 0.998).

### Total bacterial load, bacterial community richness, and community structure.

**FIG 1:**
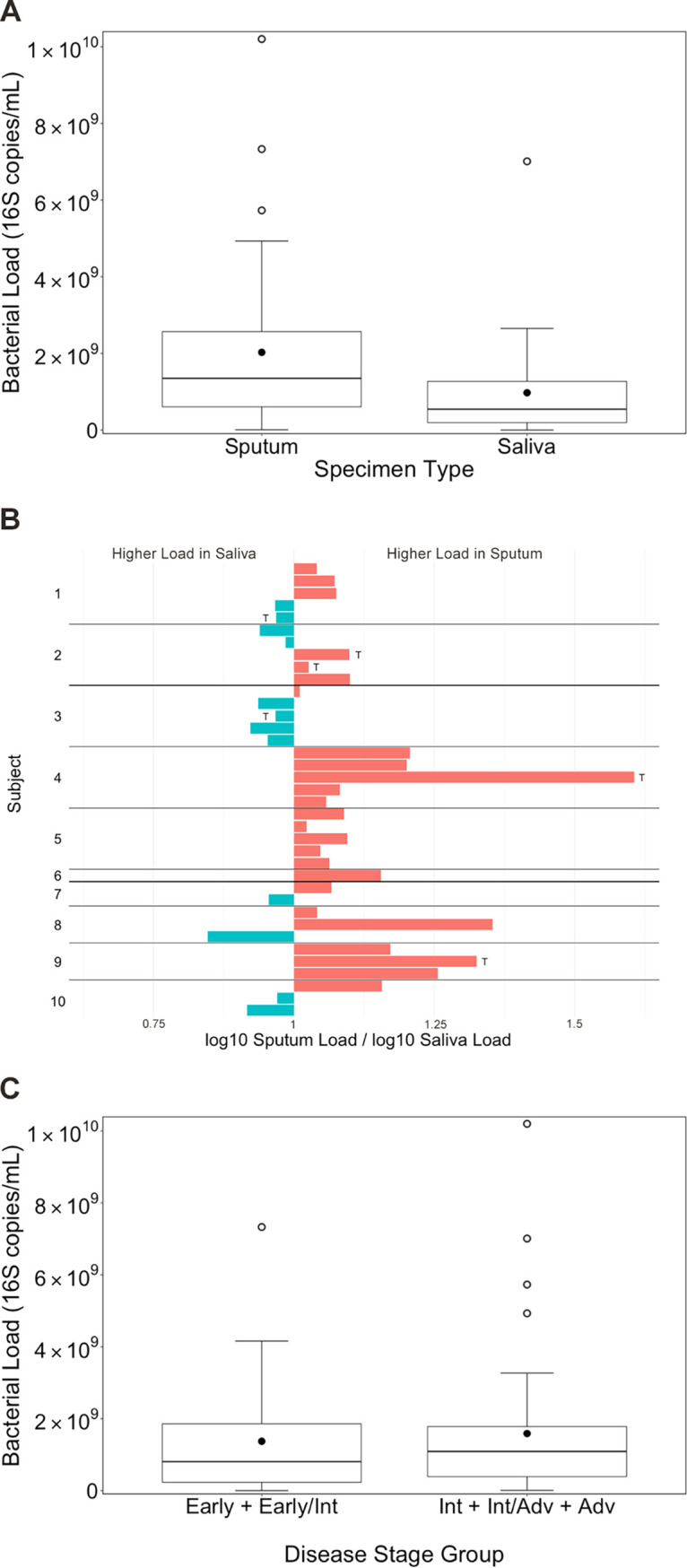
Bacterial load (16S rRNA gene copies/ml) in sputum and saliva samples. (A) In the aggregate, bacterial load was greater in sputum samples than in saliva samples, although this difference failed to reach statistical significance (linear mixed model, *P* = 0.054). (B) For 25 of 37 pairs, the bacterial load of the sputum sample was greater than that of the corresponding saliva sample. Sample pairs collected during antibiotic treatment are marked with a “T.” (C) Bacterial load of all samples (37 saliva and 37 sputum samples) was not significantly different between subjects with early or early/intermediate disease stage (*n* = 15 sample pairs from 3 subjects) and subjects with intermediate, intermediate/advanced, or advanced disease stage (*n* = 22 sample pairs from 7 subjects) (linear mixed model, *P* = 0.655). The top and bottom boundaries of each box indicate 75th and 25th quartile values, respectively; black lines inside each box represent the median values; solid black circles inside each box represent the mean values.

In the aggregate, the bacterial load (expressed as 16S rRNA gene copies/ml) in sputum samples (median 1.4 × 10^9^; range 8.3 × 10^6^ to 1.0 × 10^10^) was greater than the bacterial load in saliva samples (median 5.4 × 10^8^; range 9.3 × 10^5^ to 7.0 × 10^9^), although this difference failed to reach statistical significance (linear mixed model, *P* = 0.054; Fig. 1A). For 25 of 37 pairs, the bacterial load of the sputum sample was greater than that of the corresponding saliva sample (Fig. 1B). The median bacterial load difference between paired sputum and saliva samples was 9.3 × 10^8^ more 16S rRNA gene copies/ml in sputum than in saliva (range, 3.8 × 10^9^ fewer to 9.8 × 10^9^ more). Considering all samples (37 saliva and 37 sputum) together, bacterial load was not significantly different between disease stage groups (linear mixed model, *P* = 0.655; Fig. 1C). The bacterial load of all samples collected during antibiotic treatment was significantly lower than that of all other samples (linear mixed model, *P* = 0.038; see Table S1 in the supplemental material). Saliva samples collected during antibiotic treatment had lower bacterial load compared to nontreatment saliva samples, treatment sputum samples, and nontreatment sputum samples; however, there was no significant interaction between sample type and antibiotic treatment status in the model (linear mixed model, *P* = 0.176; Table S1).

10.1128/mSystems.00296-20.1 TABLE S1 Mean bacterial load (16S copies/ml sample) of sputum and saliva samples collected during antibiotic treatment (*n* = 6 pairs) or nontreatment samples (*n* = 31 pairs). Download Table S1, PDF file, 0.01 MB. Copyright © 2020 Lu et al. 2020 Lu et al. This content is distributed under the terms of the Creative Commons Attribution 4.0 International license.

**FIG 2:**
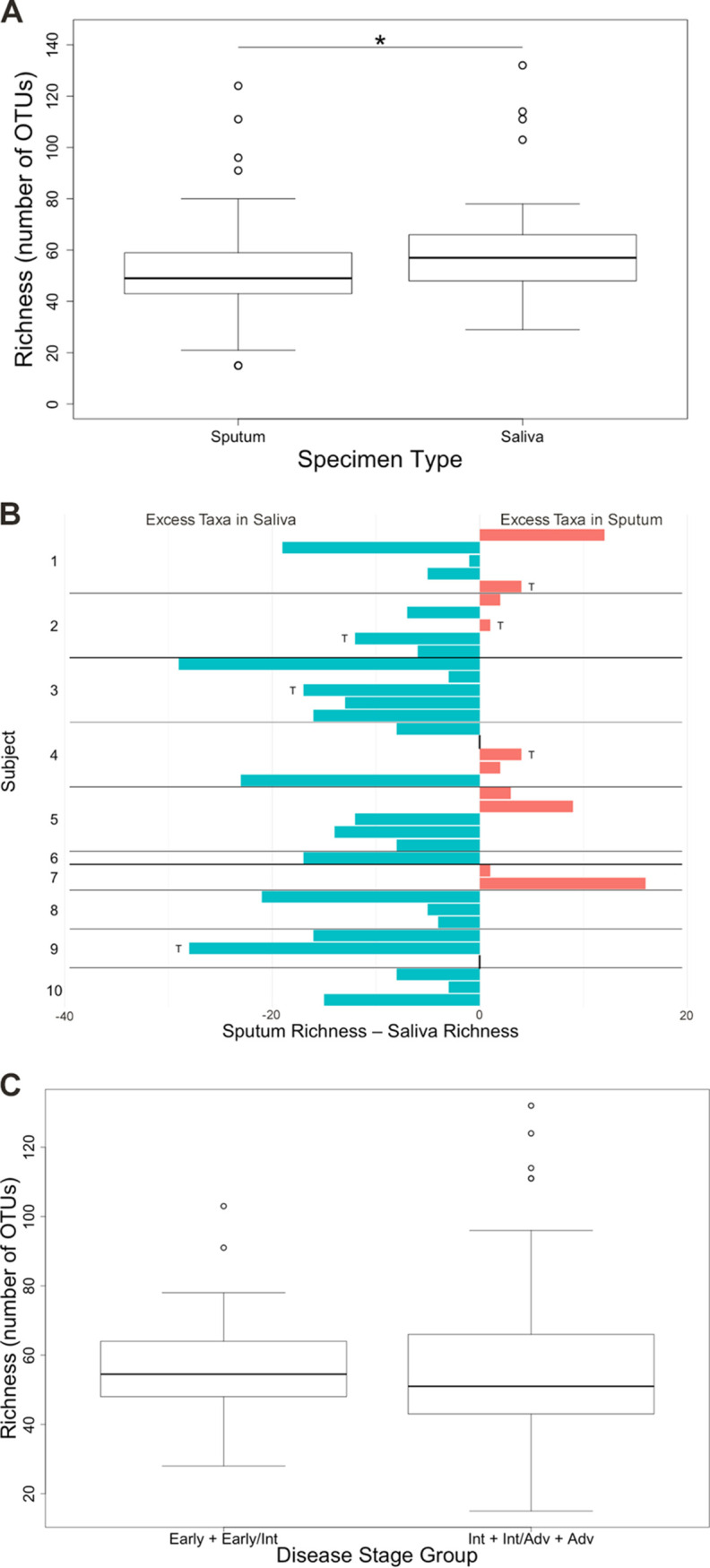
Bacterial community richness (number of observed OTUs) in sputum and saliva samples. (A) In the aggregate, bacterial richness was greater in saliva samples than in sputum samples (paired *t* test, *P* = 0.021). (B) For 25 of 37 pairs, the bacterial richness of the saliva sample was greater than that of the corresponding sputum sample. Sample pairs collected during antibiotic treatment are marked with a “T.” The vertical black bars at 0 indicate samples where sputum and saliva samples have equal richness. (C) Richness of all samples (37 saliva and 37 sputum samples) was not significantly different between subjects with early or early/intermediate disease stage (*n* = 15 sample pairs from 3 subjects) and subjects with intermediate, intermediate/advanced, or advanced disease stage (*n* = 22 sample pairs from 7 subjects). The top and bottom boundaries of each box indicate 75th and 25th quartile values, respectively; black lines inside each box represent the median values.

The median bacterial richness (number of observed operational taxonomic units [OTUs]) in sputum samples was significantly lower than in saliva (linear mixed model, *P* = 0.021; Fig. 2A). For 25 of 37 pairs, fewer taxa were observed in sputum compared to the respective saliva sample (Fig. 2B). The median difference between saliva and sputum richness was six OTUs (range, −16 to 29). For all sputum and saliva samples in the aggregate, bacterial richness was not significantly different between disease stage groups (Fig. 2C), nor between on- or off-antibiotic-treatment groups (linear mixed model, *P* = 0.958 [disease stage group] and *P* = 0.829 [antibiotic treatment groups]).

**FIG 3:**
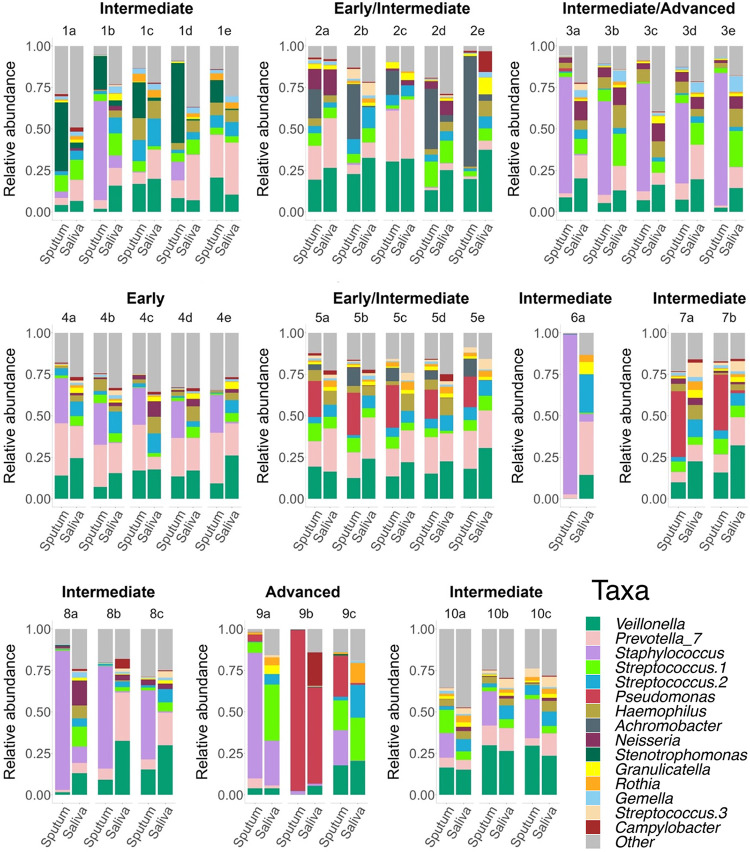
Relative abundance of taxa observed in 37 pairs of expectorated sputum and saliva samples collected from 10 subjects, showing individual OTUs with an average relative abundance >1% across all samples. “Other” taxa combine remaining OTUs in each sample. Disease stage is indicated above each subject’s plots.

**FIG 4:**
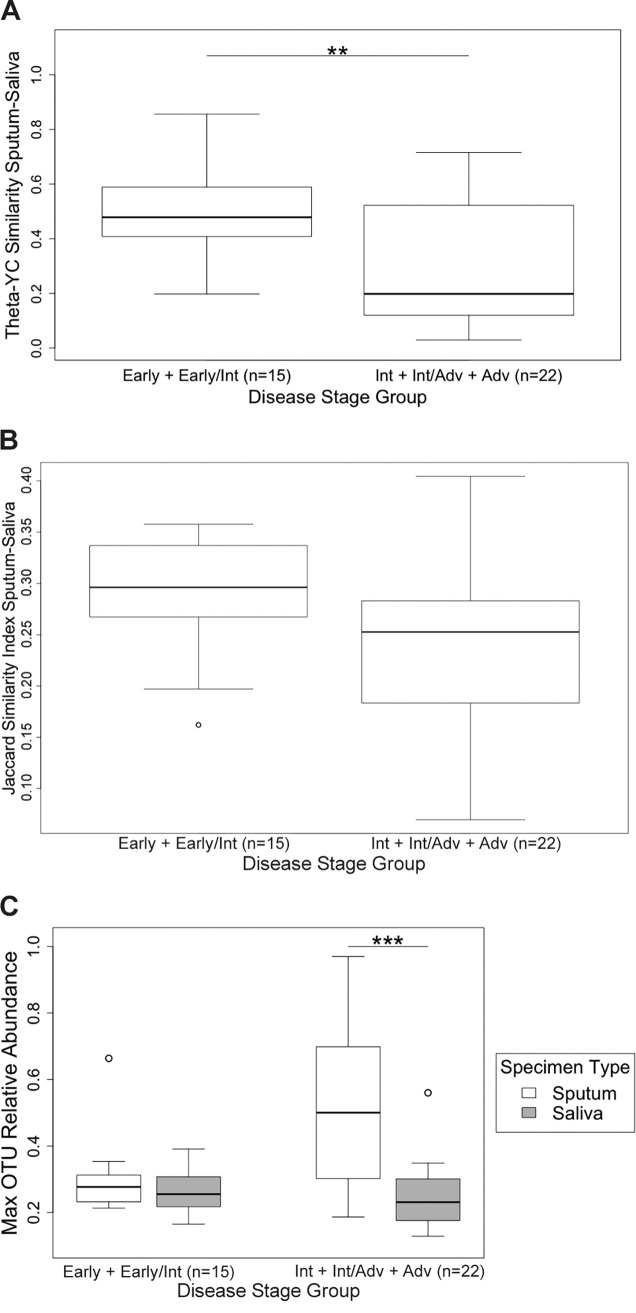
Community similarity of 37 pairs of expectorated sputum and saliva. (A) Community structures were significantly more similar (theta-YC similarity) in sample pairs from subjects with early or early/intermediate disease stage (*n* = 15 sample pairs from 3 subjects) than among subjects with intermediate, intermediate/advanced, or advanced disease stage (*n* = 22 sample pairs from 7 subjects) (*t* test, *P* = 0.007). (B) Sample pairs from subjects with later-stage disease tended to have lower Jaccard similarity than subjects with earlier-stage disease, although this difference was not statistically significant (*t* test, *P* = 0.105). (C) In sample pairs from subjects with later-stage disease, the relative abundance of the dominant (Max) OTU was significantly greater in sputum than in the respective saliva sample (paired *t* test, *P* < 0.001 [later-stage disease group] and *P* = 0.2386 [earlier-stage disease group]). The top and bottom boundaries of each box indicate 75th and 25th quartile values, respectively; black lines inside each box represent the median values.

A wide range of overlap in community membership and structure was observed in sputum-saliva sample pairs (Fig. 3). The median theta-YC similarity between each sputum and paired saliva sample was 0.400 (range 0.029 to 0.856). For context, the median theta-YC similarity between all sputum samples from all subjects was 0.207, while the median theta-YC similarity between 59 replicate generous donor control samples was 0.985. Community structures were significantly more similar for sample pairs collected from subjects at earlier (early plus early/intermediate) disease stage compared to later (intermediate plus advanced) disease stage (Fig. 4A; *t* test, *P* = 0.007). Pairs from subjects in the later disease stage group tended to have lower Jaccard similarity, although this difference was not significant (Fig. 4B; *t* test, *P* = 0.105). The relative abundance of the dominant OTU in sputum was significantly greater than the relative abundance of the dominant OTU in the respective saliva sample for sample pairs in the later disease stage group only (Fig. 4C; paired *t* tests *P* < 0.001 [later disease stage] and *P* = 0.239 [earlier disease stage]). Community structures (theta-YC and Jaccard) were not significantly different between sputum and saliva sample pairs collected during antibiotic treatment compared to all other pairs (*t* tests, *P* = 0.119 [theta-YC] and *P* = 0.979 [Jaccard]).

### *In silico* sputum decontamination.

Bacteria in sputum that originate solely via contamination from saliva during expectoration would be expected to be present in approximately the same relative proportions as are found in the respective saliva sample. In other words, regardless of the volume of saliva that contaminates a sputum sample, the taxa present in that volume of saliva would add proportionately to the sputum sample. Furthermore, the abundance of each taxon observed in a given volume of expectorated sputum that can be ascribed to saliva contamination is limited by the abundance of that taxon in a comparable volume of saliva. We used this conceptual framework to estimate the maximum possible contribution of taxa from saliva to expectorated sputum samples.

**FIG 5:**
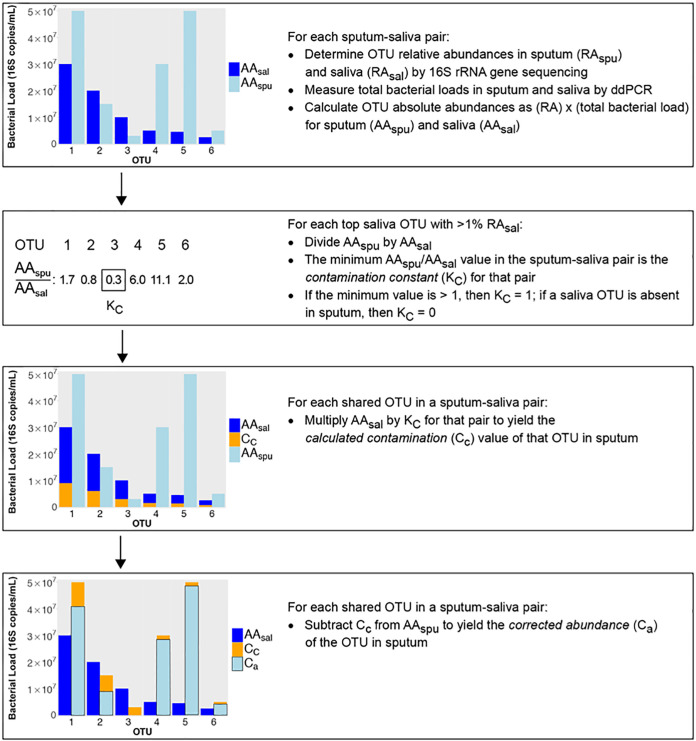
Illustration of *in silico* sputum decontamination procedure using a hypothetical pair of sputum and saliva samples.

For each sputum-saliva sample pair, the following procedure was performed, as illustrated in Fig. 5. OTUs with at least 1% relative abundance in the saliva sample (top saliva OTUs) were identified; OTUs with <1% relative abundance in the saliva sample were not included to avoid false negatives at the lower limits of sequence detection. The absolute abundance of each of the top saliva OTUs was calculated by multiplying the total bacterial load in the saliva sample (quantified by 16S rRNA droplet digital PCR [ddPCR]) by the relative abundance of the OTU in that sample. The absolute abundance of each of the top saliva OTUs present in the corresponding sputum sample was similarly calculated. The ratios of the top saliva OTUs in sputum-saliva sample pairs were calculated by dividing the absolute abundance of each of these OTUs in sputum by its absolute abundance in saliva. The lowest resulting ratio value among all top saliva OTUs in each sample pair was identified as the contamination constant for that sample pair. For pairs where the lowest resulting value was greater than 1 (i.e., the abundances of all top saliva OTUs in sputum were greater than their corresponding abundances in saliva), the contamination constant was set at 1. For sample pairs where a top saliva OTU was absent in sputum, the contamination constant for the pair was set at 0. Next, for each shared OTU in a saliva-sputum sample pair, the absolute abundance of the OTU in the saliva sample was multiplied by the contamination constant for that pair, resulting in the calculated contamination value for that OTU. This value was subtracted from the absolute abundance of that OTU in the respective sputum sample to yield a corrected abundance of the OTU in sputum. The resulting community structure, based on the corrected abundances of all shared OTUs, is the corrected community in the sputum sample.

**FIG 6:**
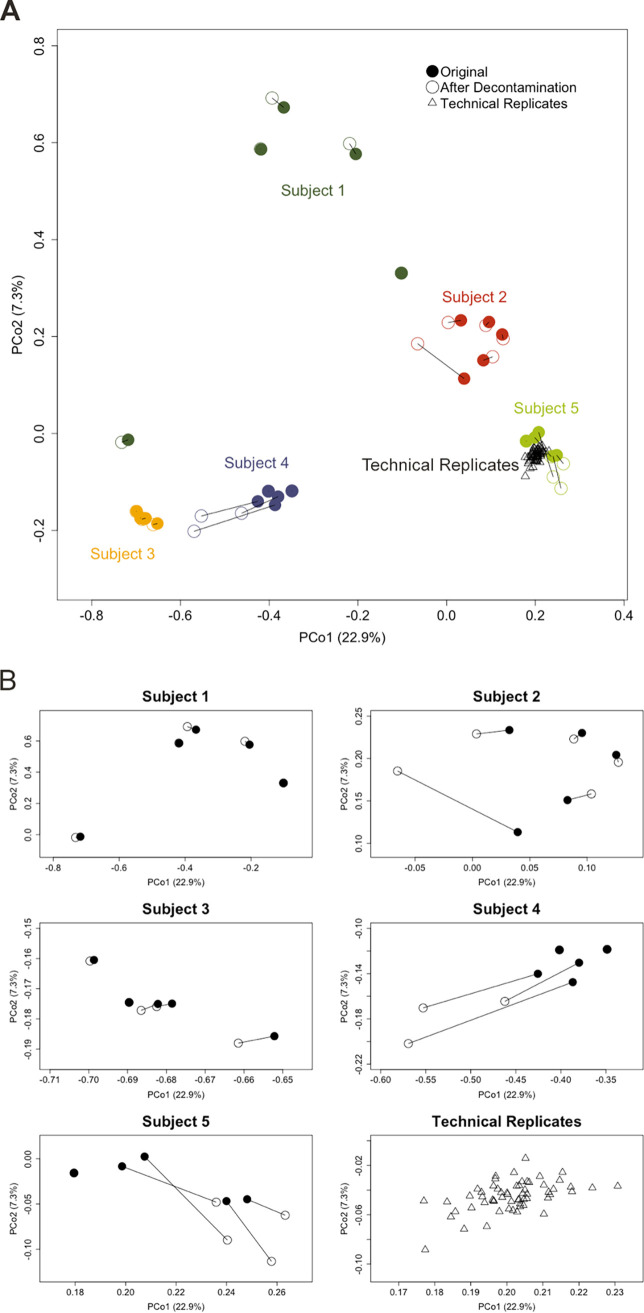
(A) Theta-YC based PCoA of bacterial community structures in sputum from five subjects who each provided five pairs of samples. Sputum before (closed circles) and after (open circles) *in silico* decontamination procedure is shown for each sample (before/after decontamination sputum pairs are connected by lines). Subjects are depicted in different colors. Fifty-nine technical replicates (generous donor controls) are shown as open gray triangles. (B) Using the same ordination data as in panel A, subjects and controls are visualized separately with the axes for each plot set so that the samples fill the ordination space. As a result, the scales between plots are not comparable.

The mean contamination constant among the 37 sputum-saliva sample pairs was 0.230 (range 0 to 1) (Table S2). The mean theta-YC similarity of pairs of sputum samples (each pair consisting of a sample pre- and post-*in silico* decontamination) was 0.970 (range 0.309 to 1). There was no significant effect of disease stage group, antibiotic treatment status, sputum alpha diversity, or sputum bacterial load on the similarity of sputum before and after decontamination (linear mixed model, *P* = 0.375 [disease stage group], *P* = 0.589 [antibiotic treatment status], *P* = 0.427 [sputum alpha diversity], and *P* = 0.886 [sputum bacterial load]). For the five subjects who each contributed five pairs of samples, the decontamination procedure did not significantly change the bacterial community structure in sputum samples (Fig. 6, analysis of molecular variance [AMOVA] *P* > 0.01).

10.1128/mSystems.00296-20.2 TABLE S2 Results of *in silico* sputum decontamination. Samples collected during antibiotic treatment are marked with a “T.” The minimum ratio of sputum absolute abundance to saliva absolute abundance is shown in column 2; this corresponds to the maximal volume of saliva per milliliter in the sputum sample. Values over 1 are in bold and considered to be 1 for the purposes of calculating contamination (i.e., contamination constant [column 3] was set at 1). For each sputum sample, the theta-YC similarity between the original community and decontaminated community is shown in column 4; similarities below 0.931 (minimum of replicate controls) are in bold. Download Table S2, PDF file, 0.02 MB. Copyright © 2020 Lu et al. 2020 Lu et al. This content is distributed under the terms of the Creative Commons Attribution 4.0 International license.

## DISCUSSION

Although chronic lung infection and inflammation is the primary cause of morbidity and mortality in CF, the microbiology of CF lung infection is not well understood. Whereas culture-based methods to study CF airway microbiology have focused on a narrow set of opportunistic bacterial pathogens, culture-independent analyses of CF respiratory specimens have revealed bacterial communities that can be quite diverse (11–15). Expectorated sputum is most commonly used in these studies, but the reliability of this specimen in reflecting lower airway microbiota is a concern, considering that expectoration involves passage of sputum through the nonsterile upper airway and oral cavity.

Few studies have addressed this concern directly. Goddard and colleagues (16) used sterile technique to obtain endobronchial samples from the airways of explanted CF lungs from several patients undergoing lung transplantation, thereby circumventing the possibility of sample contamination by upper airway secretions. Using DNA sequencing, they compared the bacterial species detected in lower respiratory tract airways to the species found in paired upper airway samples from the same patients and observed airway communities with very low diversity, heavily dominated by “typical” CF pathogens. The paucity of oropharyngeal microbiota in the endobronchial samples led the authors to question the reliability of expectorated sputum in reflecting the lower airway microbiome in CF. As the authors acknowledge, however, analysis of explanted lungs is, by definition, representative of end-stage CF lung disease, wherein very low bacterial community diversity, owing to the overwhelming dominance of a single species, is typically observed (12, 13, 17). Thus, the relevance of this study to airway microbiology in the majority of CF patients who are at earlier stages of lung disease is limited. In a subsequent investigation, Brown and colleagues (18) dissected the diseased lung tissue of a 3-year-old child who underwent lobectomy for severe localized CF lung disease and similarly investigated the microbiota therein. In addition to typical CF pathogens, including *Staphylococcus* and *Haemophilus*, a diverse bacterial population was detected that included *Ralstonia* and anaerobic species.

More recently, Jorth and colleagues (4) reported that DNA sequence analysis of BAL fluid samples from children and young adults with mild CF lung disease showed no evidence of oral bacterial communities in samples that lacked typical CF pathogens. They concluded that established CF pathogens are primarily responsible for CF lung infections. The BAL fluid samples from these relatively healthy young CF patients (mean ppFEV_1_ of 92; mean age 13 years; median age not provided) generally had bacterial DNA concentrations (median ∼10^3^ bacterial genome copies/ml) several orders of magnitude lower than the levels found in sputum of expectorating adults with CF (12, 19–21), making it difficult to extrapolate these findings more broadly to the CF population.

In our study, we similarly observed bacterial DNA concentrations in sputum (median ∼10^9^ 16S gene copies/ml) far exceeding those measured by Jorth and colleagues (4) in BAL fluid samples from younger patients. Whereas sample contamination introduced by laboratory reagents and/or during sample collection is a critically important concern in microbiome analyses of low-biomass samples (22–24), the impact would be expected to be considerably less in the analysis of samples with very large biomass. In fact, we observed that bacterial DNA levels in our reagent controls were several logs lower than those found in sputum and saliva samples. More relevant to our study, however, was our observation that bacterial loads in sputum were generally greater than those in saliva (Fig. 1A and B), which would considerably mitigate the impact of salivary contamination of expectorated sputum relative to what would be expected with salivary contamination of very-low-biomass BAL fluid samples. Consistent with previous work (25), we found that antibiotic treatment was not a significant factor contributing to differences in bacterial load or bacterial richness.

Our analyses of bacterial community structures revealed considerable overlaps in membership within pairs of sputum and saliva samples. This is not surprising considering that sputum and saliva samples each likely represent a mixture of the two. Nevertheless, detecting taxa in sputum that are known to inhabit the upper airways and oropharynx raises reasonable concerns about the source of these species. Assessing lower airway infection in CF from an ecological theory perspective, however, provides a cogent conceptual framework whereby the upper airway serves as a source of microbes that may migrate to and persist in the lower airways (26). Microbiologic continuity of the aerodigestive tract has been identified in healthy individuals (27) as well as in persons with CF (28), where similarity between oral and gastric communities indicates connectivity between mouth, lung, and upper gastrointestinal microbiomes. In this biogeography context, continuously mixed microbial communities inhabit these contiguous anatomic sites, with community composition in each determined by rates of migration, colonization/infection, and extinction (26, 29).

As has been shown in several previous studies (12, 13, 17), we found that sputum bacterial community diversity decreased with lung disease progression, primarily due to reduced community evenness resulting from the progressive dominance of a single species. This trend was not observed in the corresponding saliva samples, however. Further, we found, as have others (30), that sputum and saliva bacterial loads remained relatively constant with advancing lung disease. Given this consistency in bacterial load and salivary community diversity across disease stages, it is improbable that salivary contamination would account for high sputum community diversity during early stages of lung disease but not during late disease stages.

We found that despite the overlap in bacterial community membership between paired sputum and saliva samples, we observed, in some pairs, taxa that were disproportionately represented in one sample or the other. We noted, for example, some sample pairs with comparable bacterial loads in which a taxon was present in high relative abundance in saliva but present in very low relative abundance or not detected at all in the corresponding sputum sample. Conversely, we observed some sample pairs in which a specific taxon was present in much greater relative abundance in sputum than in the corresponding saliva sample. Our *in silico* decontamination model is premised on the principle that the contribution of species from saliva to sputum must be proportionate to the relative abundance of species in saliva. Taking total bacterial load and taxon relative abundance into account in this model allows an estimation of the maximum salivary contamination of sputum for each sample pair, providing a liberal estimate of potential contamination and a means to “decontaminate” expectorated sputum *in silico*.

While the thrust of our study was to estimate the impact of salivary microbiota on measures of microbial communities in sputum, this effort allowed us to consider previously described software packages for estimating the proportion of contaminants in microbial communities of interest. Programs such as SourceTracker (31) and decontam (32) seek to objectively remove entire OTUs or genera based on their *a priori* identification as contaminants, or their low abundance, or their presence in control samples. In contrast, our approach considers the absolute abundances of taxa and estimates contamination separately for each pair of sputum and saliva samples. This strategy is more similar to microDecon (33), which also addresses overlapping OTUs (those OTUs expected to occur in both the sample and the contamination source) based on the principle that contamination from a common source will be proportionate and can be calculated using a “constant” OTU that is assumed to be entirely contamination. The difference of our approach is that for each sample and source pair, we use the estimation of maximum contamination to establish an upper limit of contamination, rather than make a probabilistic estimation of contamination (SourceTracker) or remove taxa entirely (decontam). The primary goal of our method was to evaluate the potential for saliva contamination of expectorated sputum, which in turn establishes the utility of sputum as a measure of lower airway microbiota in CF. The calculated saliva contamination of sputum samples therefore represents a “worst-case scenario.”

Despite this aggressive approach, the impact of saliva contamination on sputum samples was estimated to be minimal. In fact, the mean sputum community structure similarity before and after decontamination was within the range of technical (sequencing) replicate controls, with only three of 37 sputum samples falling outside this range, suggesting that much of the diversity observed in sputum (i.e., due to nontypical CF pathogens) was present prior to passage through the oral cavity during expectoration. While we do not think *in silico* decontamination should be a necessary step for all CF microbiome studies employing expectorated sputum, it is nevertheless possible that even the minor changes in community structures observed after decontamination may reveal dynamics of interest that would have been otherwise obscured. In subject 5, for example, the bacterial community structures of four of five sputum samples shifted in a similar fashion after *in silico* decontamination. In this regard, the impact of salivary contamination on community structure—and whether this constitutes a meaningful change—likely varies between subjects and over time within subjects and ultimately depends on the question at hand. Importantly, this study provides evidence that the diversity of bacterial communities measured in CF sputum cannot be attributed solely to the contamination of sputum by salivary microbiota during expectoration. As such, these results support the use of expectorated sputum as a measure of lower airway microbiota in CF and do not corroborate reports asserting that CF lung microbial diversity is predominantly an artifact of sampling methods.

## MATERIALS AND METHODS

### Study design and sample collection.

Expectorated sputum and saliva samples were collected from a cohort of persons with CF as part of a long-term study of CF airway microbiota. This study was approved by the Institutional Review Board of the University of Michigan Medical School (HUM00037056), and informed written consent was obtained from all participants. Sputum and saliva samples were collected by subjects at home in 30-ml sterile conical tubes. Subjects were reminded that sputum was the thick material from deep in the lungs and not the watery fluid in their mouth. Subjects were instructed to spit out saliva and rinse their mouth with tap water before taking a deep breath and coughing deeply to move sputum up from their lungs. Sputum was then expectorated directly into a collection tube. For saliva samples, subjects were asked not to eat or drink for 30 min prior to collection. Subjects were instructed to allow saliva to collect in their mouths for at least 1 min and then gently spit or drool into the collection tubes. Samples were immediately stored in a −20°C manual defrost freezer (1 to 10 weeks; mean 4 weeks) before same-day courier transport on dry ice to the University of Michigan and immediate storage at −80°C. We have previously shown that sputum storage at −20°C does not have a significant effect on sequencing-based measures of bacterial community structure (34).

Electronic medical records were reviewed for subject demographic and clinical data. Daily antibiotic use was recorded by subjects on the day the samples were collected, and subjects were classified as either on or not on treatment antibiotics (i.e., antibiotics other than chronic maintenance antibiotics such as inhaled tobramycin and oral azithromycin) at the time of sample collection. Disease stage was assigned based on ppFEV_1_ over the period of sample collection: early (ppFEV_1_ > 70), intermediate (70 ≥ ppFEV_1_ ≥ 40), or advanced (ppFEV_1_ < 40) (35, 36). When subjects crossed between disease stage categories during the course of this study, they were classified as either early/intermediate or intermediate/advanced.

### Sample processing and 16S rRNA gene sequencing.

Sputum and saliva samples were thawed on ice prior to homogenization in 10% Sputolysin (MilliporeSigma, Burlington, MA, USA). Samples were treated with bacterial lysis buffer (Roche Diagnostics Corp., Indianapolis, IN, USA), lysostaphin (MilliporeSigma), and lysozyme (MilliporeSigma) as previously described (12), followed by mechanical disruption by glass bead beating and digestion with proteinase K (Qiagen Sciences, Germantown, MD, USA). The MagNA Pure nucleic acid purification platform (Roche Diagnostics Corp.) was used to extract and purify DNA according to the manufacturer’s protocol. Reagent control samples were similarly prepared, with UltraPure DNase/RNase-free distilled water (Life Technologies Corp., Grand Island, NY, USA) substituted for the sample.

DNA libraries were prepared by the University of Michigan Microbial Systems Molecular Biology Laboratory as described previously (37). In brief, the V4 region of the bacterial 16S rRNA gene was amplified using touchdown PCR with barcoded dual-index primers. The touchdown PCR cycles consisted of 2 min at 95°C, followed by 20 cycles of 95°C for 20 s, 60°C (starting from 60°C, the annealing temperature decreased 0.3°C each cycle) for 15 s, and 72°C for 5 min, followed by 20 cycles of 95°C for 20 s, 55°C for 15 s, and 72°C for 5 min and a final 72°C for 10 min. Resulting amplicon libraries were normalized and sequenced on an Illumina sequencing platform using MiSeq reagent kit V2 (Illumina Inc., San Diego, CA, USA). The final load concentration was 4.0 to 5.5 pM with a 15% PhiX spike to add diversity.

### DNA sequencing controls and DNA sequence analyses.

Reagent-only controls were prepared with each change in reagent lot. Mock bacterial community DNA standards (ZymoBIOMICS Microbial Community DNA; Zymo Research, Irvine, CA, USA) and water controls were included in sequencing runs by the University of Michigan Microbial Systems Molecular Biology Laboratory. Sequences from reagent and water controls were analyzed to assess the impact of laboratory contaminants on sputum and saliva sample DNA sequencing results. Mock community sequences were used to measure sequencing error rates. DNA extracted from a “generous donor” CF sputum sample was also sequenced to determine interrun sequencing variability. Sequencing data from 59 replicates of the generous donor control were analyzed.

Raw DNA sequences were analyzed with mothur v1.41.3 (38) following the mothur MiSeq SOP (full list of mothur commands available at https://github.com/um-cf-microbiome/LiPuma_Sputum-Saliva). Briefly, low-quality and chimeric reads were discarded, and remaining sequences were classified taxonomically against the SILVA database (release 132) using the RDP Bayesian Classifier. Sequences were clustered into OTUs using a 3% dissimilarity cutoff with the OptiClust algorithm (39). To limit the effects of sequencing depth, each sample was rarefied to the lowest number of reads in the sample set (*n* = 2,076). Alpha diversity of the subsampled data was measured as observed OTUs (richness). Beta diversity was calculated based on the Jaccard (40) or Yue and Clayton (“theta-YC”) (41) measures of dissimilarity and, for ease of interpretation, are reported as similarity (i.e., 1 − dissimilarity). Theta-YC was chosen to describe dissimilarity between communities, taking into consideration the proportions of both the shared and nonshared members of each community. An advantage of this metric compared to other dissimilarity metrics is that it is less sensitive to outlier values and weighs rare and abundant OTUs more evenly than the Bray-Curtis or Morisita-Horn (42) metric.

### ddPCR.

Total bacterial load of sample and control DNAs was quantified by 16S rRNA droplet digital PCR (ddPCR) on a QX200 AutoDG droplet digital PCR system (Bio-Rad Laboratories, Inc., Hercules, CA, USA). Primer sequences were 5′- TCCTACGGGAGGCAGCAGT-3′ and 5′- GGACTACCAGGGTATCTAATCCTG-3′ (final concentration 900 nM each), and probe sequence was (6-carboxyfluorescein [FAM])-5′- CGTATTACCGCGGCTGCTGG-3′-(6-carboxytetramethylrhodamine [TAMRA]) (final concentration 250 nM). All reactions were run in duplicate. Reaction mixtures were transferred to an automated droplet generator (Bio-Rad Laboratories, Inc.), followed by gene amplification in a C1000 Touch thermal cycler (Bio-Rad Laboratories, Inc.). Cycling conditions were 10 min at 95°C, followed by 40 cycles at 94°C for 30 s and 58°C for 2 min, and a final 98°C for 10 min, with a ramp rate of 2°C/s per step. DNA quantification was performed with the QX200 droplet reader (Bio-Rad) and data analysis with QuantaSoft Analysis Pro (Bio-Rad) using default parameters for threshold amplification. Reaction mixtures with fewer than 10,000 droplets were omitted from analysis. DNA concentrations of replicates were averaged, adjusted for dilution factor, reported in copies of target gene per microliter of DNA, and then converted to copies of target gene per milliliter of sample based on the DNA extraction steps.

### Data analyses.

A linear mixed effects model was used to compare bacterial load between sputum and saliva samples (R packages ‘lme4’ [43] and ‘lmerTest’ [44]), with bacterial load as the dependent variable; sample type, disease stage group, antibiotic treatment status, and an interaction term for antibiotic treatment and sample type as fixed effects; and intercept for subject as a random effect. A linear mixed effects model was also used to compare bacterial richness between sputum and saliva samples, with richness (number of observed OTUs) as the dependent variable; sample type, disease stage group, and antibiotic treatment status as fixed effects; and intercept for subject as a random effect. *t* tests were performed to compare sputum-saliva theta-YC and Jaccard similarities between disease stage groups and between antibiotic treatment status groups. Paired *t* tests were performed to compare dominant OTU (the OTU with the highest relative abundance in the sample) relative abundances between sample types within disease stage groups.

A theta-YC-based principal-coordinate analysis (PCoA) plot was generated to visualize the effects of *in silico* saliva decontamination on sputum samples and to compare these effects to repeat sequencing of a generous donor control sample. Analysis of molecular variance (AMOVA) was used to compare differences in centroids of sputum bacterial communities within individual subjects before and after decontamination (mothur, v1.41.3). A Bonferroni-corrected *P* value of 0.01 was used to assess significance. A linear mixed effects model was used to model the impact of sample variables on the effect of the sputum decontamination procedure, with theta-YC similarity of sputum before and after decontamination as the dependent variable; sputum alpha diversity (as inverse Simpson index), sputum bacterial load, disease stage group, and antibiotic treatment status as fixed effects; and intercept for subject as a random effect.

### Data availability.

Raw sequence data are available at NCBI (SRA accession no. PRJNA611611). Detailed protocols, mothur log file, deidentified subject data, 16S rRNA gene sequencing data, 16S ddPCR data, and reproducible code for data analyses and figures are available on GitHub (https://github.com/um-cf-microbiome/LiPuma_Sputum-Saliva).

## References

[1] LiPuma JJ 2010 The changing microbial epidemiology in cystic fibrosis. Clin Microbiol Rev 23:299–323. doi:10.1128/CMR.00068-09. 20375354 PMC2863368

[2] Huang YJ, LiPuma JJ 2016 The microbiome in cystic fibrosis. Clin Chest Med 37:59–67. doi:10.1016/j.ccm.2015.10.003. 26857768 PMC5154676

[3] O’Toole GA 2018 Cystic fibrosis airway microbiome: overturning the old, opening the way for the new. J Bacteriol 200:e00561-17. doi:10.1128/JB.00561-17. 29084859 PMC5786703

[4] Jorth P, Ehsan Z, Rezayat A, Caldwell E, Pope C, Brewington JJ, Goss CH, Benscoter D, Clancy JP, Singh PK 2019 Direct lung sampling indicates that established pathogens dominate early infections in children with cystic fibrosis. Cell Rep 27:1190–1204. doi:10.1016/j.celrep.2019.03.086. 31018133 PMC6668708

[5] Hogan DA, Willger SD, Dolben EL, Hampton TH, Stanton BA, Morrison HG, Sogin ML, Czum J, Ashare A 2016 Analysis of lung microbiota in bronchoalveolar lavage, protected brush and sputum samples from subjects with mild-to-moderate cystic fibrosis lung disease. PLoS One 11:e0149998. doi:10.1371/journal.pone.0149998. 26943329 PMC4778801

[6] Muhlebach MS, Zorn BT, Esther CR, Hatch JE, Murray CP, Turkovic L, Ranganathan SC, Boucher RC, Stick SM, Wolfgang MC 2018 Initial acquisition and succession of the cystic fibrosis lung microbiome is associated with disease progression in infants and preschool children. PLoS Pathog 14:e1006798. doi:10.1371/journal.ppat.1006798. 29346420 PMC5773228

[7] Frayman KB, Armstrong DS, Carzino R, Ferkol TW, Grimwood K, Storch GA, Teo SM, Wylie KM, Ranganathan SC 2017 The lower airway microbiota in early cystic fibrosis lung disease: a longitudinal analysis. Thorax 72:1104–1112. doi:10.1136/thoraxjnl-2016-209279. 28280235

[8] Pittman JE, Wylie KM, Akers K, Storch GA, Hatch J, Quante J, Frayman KB, Clarke N, Davis M, Stick SM, Hall GL, Montgomery G, Ranganathan S, Davis SD, Ferkol TW, Australian Respiratory Early Surveillance Team for Cystic Fibrosis. 2017 Association of antibiotics, airway microbiome, and inflammation in infants with cystic fibrosis. Ann Am Thorac Soc 14:1548–1555. doi:10.1513/AnnalsATS.201702-121OC. 28708417 PMC5718571

[9] Renwick J, McNally P, John B, DeSantis T, Linnane B, Murphy P, SHIELD CF. 2014 The microbial community of the cystic fibrosis airway is disrupted in early life. PLoS One 9:e109798. doi:10.1371/journal.pone.0109798. 25526264 PMC4272276

[10] Willner D, Haynes MR, Furlan M, Schmieder R, Lim YW, Rainey PB, Rohwer F, Conrad D 2012 Spatial distribution of microbial communities in the cystic fibrosis lung. ISME J 6:471–474. doi:10.1038/ismej.2011.104. 21796216 PMC3260497

[11] Cox MJ, Allgaier M, Taylor B, Baek MS, Huang YJ, Daly RA, Karaoz U, Andersen GL, Brown R, Fujimura KE, Wu B, Tran D, Koff J, Kleinhenz ME, Nielson D, Brodie EL, Lynch SV 2010 Airway microbiota and pathogen abundance in age-stratified cystic fibrosis patients. PLoS One 5:e11044. doi:10.1371/journal.pone.0011044. 20585638 PMC2890402

[12] Zhao J, Schloss PD, Kalikin LM, Carmody LA, Foster BK, Petrosino JF, Cavalcoli JD, VanDevanter DR, Murray S, Li JZ, Young VB, LiPuma JJ 2012 Decade-long bacterial community dynamics in cystic fibrosis airways. Proc Natl Acad Sci U S A 109:5809–5814. doi:10.1073/pnas.1120577109. 22451929 PMC3326496

[13] Coburn B, Wang PW, Diaz Caballero J, Clark ST, Brahma V, Donaldson S, Zhang Y, Surendra A, Gong Y, Tullis ED, Yau YC, Waters VJ, Hwang DM, Guttman DS 2015 Lung microbiota across age and disease stage in cystic fibrosis. Sci Rep 5:10241. doi:10.1038/srep10241. 25974282 PMC4431465

[14] Whelan FJ, Heirali AA, Rossi L, Rabin HR, Parkins MD, Surette MG 2017 Longitudinal sampling of the lung microbiota in individuals with cystic fibrosis. PLoS One 12:e0172811. doi:10.1371/journal.pone.0172811. 28253277 PMC5333848

[15] Lucas SK, Yang R, Dunitz JM, Boyer HC, Hunter RC 2018 16S rRNA gene sequencing reveals site-specific signatures of the upper and lower airways of cystic fibrosis patients. J Cyst Fibros 17:204–212. doi:10.1016/j.jcf.2017.08.007. 28826586 PMC5817045

[16] Goddard AF, Staudinger BJ, Dowd SE, Joshi-Datar A, Wolcott RD, Aitken ML, Fligner CL, Singh PK 2012 Direct sampling of cystic fibrosis lungs indicates that DNA-based analyses of upper-airway specimens can misrepresent lung microbiota. Proc Natl Acad Sci U S A 109:13769–13774. doi:10.1073/pnas.1107435109. 22872870 PMC3427132

[17] Carmody LA, Caverly LJ, Foster BK, Rogers MAM, Kalikin LM, Simon RH, VanDevanter DR, LiPuma JJ 2018 Fluctuations in airway bacterial communities associated with clinical states and disease stages in cystic fibrosis. PLoS One 13:e0194060. doi:10.1371/journal.pone.0194060. 29522532 PMC5844593

[18] Brown PS, Pope CE, Marsh RL, Qin X, McNamara S, Gibson R, Burns JL, Deutsch G, Hoffman LR 2014 Directly sampling the lung of a young child with cystic fibrosis reveals diverse microbiota. Ann Am Thorac Soc 11:1049–1055. doi:10.1513/AnnalsATS.201311-383OC. 25072206 PMC4214062

[19] Stressmann FA, Rogers GB, Marsh P, Lilley AK, Daniels TWV, Carroll MP, Hoffman LR, Jones G, Allen CE, Patel N, Forbes B, Tuck A, Bruce KD 2011 Does bacterial density in cystic fibrosis sputum increase prior to pulmonary exacerbation? J Cyst Fibros 10:357–365. doi:10.1016/j.jcf.2011.05.002. 21664196

[20] Price KE, Hampton TH, Gifford AH, Dolben EL, Hogan DA, Morrison HG, Sogin ML, O’Toole GA 2013 Unique microbial communities persist in individual cystic fibrosis patients throughout a clinical exacerbation. Microbiome 1:27. doi:10.1186/2049-2618-1-27. 24451123 PMC3971630

[21] Zemanick ET, Harris JK, Wagner BD, Robertson CE, Sagel SD, Stevens MJ, Accurso FJ, Laguna TA 2013 Inflammation and airway microbiota during cystic fibrosis pulmonary exacerbations. PLoS One 8:e62917. doi:10.1371/journal.pone.0062917. 23646159 PMC3639911

[22] Salter SJ, Cox MJ, Turek EM, Calus ST, Cookson WO, Moffatt MF, Turner P, Parkhill J, Loman NJ, Walker AW 2014 Reagent and laboratory contamination can critically impact sequence-based microbiome analyses. BMC Biol 12:87. doi:10.1186/s12915-014-0087-z. 25387460 PMC4228153

[23] Kim D, Hofstaedter CE, Zhao C, Mattei L, Tanes C, Clarke E, Lauder A, Sherrill-Mix S, Chehoud C, Kelsen J, Conrad M, Collman RG, Baldassano R, Bushman FD, Bittinger K 2017 Optimizing methods and dodging pitfalls in microbiome research. Microbiome 5:52. doi:10.1186/s40168-017-0267-5. 28476139 PMC5420141

[24] Eisenhofer R, Minich JJ, Marotz C, Cooper A, Knight R, Weyrich LS 2019 Contamination in low microbial biomass microbiome studies: issues and recommendations. Trends Microbiol 27:105–117. doi:10.1016/j.tim.2018.11.003. 30497919

[25] Fodor AA, Klem ER, Gilpin DF, Elborn JS, Boucher RC, Tunney MM, Wolfgang MC 2012 The adult cystic fibrosis airway microbiota is stable over time and infection type, and highly resilient to antibiotic treatment of exacerbations. PLoS One 7:e45001. doi:10.1371/journal.pone.0045001. 23049765 PMC3458854

[26] Whiteson KL, Bailey B, Bergkessel M, Conrad D, Delhaes L, Felts B, Harris JK, Hunter R, Lim YW, Maughan H, Quinn R, Salamon P, Sullivan J, Wagner BD, Rainey PB 2014 The upper respiratory tract as a microbial source for pulmonary infections in cystic fibrosis. Parallels from island biogeography. Am J Respir Crit Care Med 189:1309–1315. doi:10.1164/rccm.201312-2129PP. 24702670 PMC4098084

[27] Bassis CM, Erb-Downward JR, Dickson RP, Freeman CM, Schmidt TM, Young VB, Beck JM, Curtis JL, Huffnagle GB 2015 Analysis of the upper respiratory tract microbiotas as the source of the lung and gastric microbiotas in healthy individuals. mBio 6:e00037-15. doi:10.1128/mBio.00037-15. 25736890 PMC4358017

[28] Al-Momani H, Perry A, Stewart CJ, Jones R, Krishnan A, Robertson AG, Bourke S, Doe S, Cummings SP, Anderson A, Forrest T, Griffin SM, Brodlie M, Pearson J, Ward C 2016 Microbiological profiles of sputum and gastric juice aspirates in cystic fibrosis patients. Sci Rep 6:26985. doi:10.1038/srep26985. 27245316 PMC4887896

[29] Venkataraman A, Bassis CM, Beck JM, Young VB, Curtis JL, Huffnagle GB, Schmidt TM 2015 Application of a neutral community model to assess structuring of the human lung microbiome. mBio 6:e02284-14. doi:10.1128/mBio.02284-14. 25604788 PMC4324308

[30] Zemanick ET, Wagner BD, Robertson CE, Stevens MJ, Szefler SJ, Accurso FJ, Sagel SD, Harris JK 2015 Assessment of airway microbiota and inflammation in cystic fibrosis using multiple sampling methods. Ann Am Thorac Soc 12:221–229. doi:10.1513/AnnalsATS.201407-310OC. 25474078 PMC4342834

[31] Knights D, Kuczynski J, Charlson ES, Zaneveld J, Mozer MC, Collman RG, Bushman FD, Knight R, Kelley ST 2011 Bayesian community-wide culture-independent microbial source tracking. Nat Methods 8:761–763. doi:10.1038/nmeth.1650. 21765408 PMC3791591

[32] Davis NM, Proctor DM, Holmes SP, Relman DA, Callahan BJ 2018 Simple statistical identification and removal of contaminant sequences in marker-gene and metagenomics data. Microbiome 6:226. doi:10.1186/s40168-018-0605-2. 30558668 PMC6298009

[33] McKnight DT, Huerlimann R, Bower DS, Schwarzkopf L, Alford RA, Zenger KR 2019 microDecon: a highly accurate read‐subtraction tool for the post‐sequencing removal of contamination in metabarcoding studies. Environ DNA 1:14–25. doi:10.1002/edn3.11.

[34] Zhao J, Li J, Schloss PD, Kalikin LM, Raymond TA, Petrosino JF, Young VB, LiPuma JJ 2011 Effect of sample storage conditions on culture-independent bacterial community measures in cystic fibrosis sputum specimens. J Clin Microbiol 49:3717–3718. doi:10.1128/JCM.01189-11. 21865433 PMC3187318

[35] Carmody LA, Zhao J, Schloss PD, Petrosino JF, Murray S, Young VB, Li JZ, LiPuma JJ 2013 Changes in cystic fibrosis airway microbiota at pulmonary exacerbation. Ann Am Thorac Soc 10:179–187. doi:10.1513/AnnalsATS.201211-107OC. 23802813 PMC3960905

[36] Konstan MW, Wagener JS, VanDevanter DR 2009 Characterizing aggressiveness and predicting future progression of CF lung disease. J Cystic Fibrosis 8:S15–19. doi:10.1016/S1569-1993(09)60006-0. PMC4167362 19460682

[37] Seekatz AM, Theriot CM, Molloy CT, Wozniak KL, Bergin IL, Young VB 2015 Fecal microbiota transplantation eliminates *Clostridium difficile* in a murine model of relapsing disease. Infect Immun 83:3838–3846. doi:10.1128/IAI.00459-15. 26169276 PMC4567621

[38] Kozich JJ, Westcott SL, Baxter NT, Highlander SK, Schloss PD 2013 Development of a dual-index sequencing strategy and curation pipeline for analyzing amplicon sequence data on the MiSeq Illumina sequencing platform. Appl Environ Microbiol 79:5112–5120. doi:10.1128/AEM.01043-13. 23793624 PMC3753973

[39] Westcott SL, Schloss PD 2017 OptiClust, an improved method for assigning amplicon-based sequence data to operational taxonomic units. mSphere 2:e00073-17. doi:10.1128/mSphereDirect.00073-17. 28289728 PMC5343174

[40] Jaccard P 1908 Nouvelles recherches sur la distribution florale. Bull Soc Vaudoise Sci Nat 44:223–270.

[41] Yue JC, Murray KC 2005 A similarity measure based on species proportions. Commun Stat Theory Methods 34:2123–2131. doi:10.1080/STA-200066418.

[42] Schloss PD, Gevers D, Westcott SL 2011 Reducing the effects of PCR amplification and sequencing artifacts on 16S rRNA-based studies. PLoS One 6:e27310. doi:10.1371/journal.pone.0027310. 22194782 PMC3237409

[43] Bates D, Maechler M, Bolker B, Walker S 2015 Fitting linear mixed-effects models using lme4. J Stat Softw 67:1–48.

[44] Kuznetsova A, Brockhoff PB, Christensen R 2017 lmerTest package: tests in linear mixed effects models. J Stat Softw 82:1–26.

