# Supplementary material for: Parallel Analysis of Cystic Fibrosis Sputum and Saliva Reveals Overlapping Communities and an Opportunity for Sample Decontamination
Source: mSystems. 2020 Jul 7;5(4):e00296-20. doi: 10.1128/mSystems.00296-20 (PMC7343308; doi:10.1128/mSystems.00296-20)
Supplement: TABLE S1 [file mSystems.00296-20-st001.pdf]

Table S1

Sample Type	Mean (range) Bacterial Load (16S copies/mL sample)	
	**Treatment (n=6 pairs)**	**Non-Treatment (n=31 pairs)**
Saliva	7.7×10^{8} (9.3×10^{5} – 2.6×10^{9})	1.0×10^{9} (7.2×10^{6} – 7.0×10^{9})
Sputum	1.5×10^{9} (8.3×10^{6} – 3.8×10^{9})	2.1×10^{9} (5.1×10^{7} – 1.0×10^{10})
**All Samples**	1.1×10^{9} (9.3×10^{5} – 3.8×10^{9})	1.6×10^{9} (7.2×10^{6} – 1.0×10^{10})
