# Supplementary material for: Parallel Analysis of Cystic Fibrosis Sputum and Saliva Reveals Overlapping Communities and an Opportunity for Sample Decontamination
Source: mSystems. 2020 Jul 7;5(4):e00296-20. doi: 10.1128/mSystems.00296-20 (PMC7343308; doi:10.1128/mSystems.00296-20)
Supplement: TABLE S2 [file mSystems.00296-20-st002.pdf]

Table S2

Pairs	Minimum Sputum AA/Saliva AA	Contamination Constant	Theta-YC Similarity
**1a**	0.282	0.282	0.995
**1b**	0.212	0.212	1
**1c**	0.103	0.103	0.999
**1d**	0.008	0.008	1
**1e (T)**	0.002	0.002	1
**2a**	0.184	0.184	**0.309**
**2b**	0.234	0.234	**0.913**
**2c (T)**	**2.130**	1	**0.858**
**2d (T)**	0.271	0.271	0.980
**2e**	0.640	0.640	0.997
**3a**	0.046	0.046	1
**3b**	0.039	0.039	0.993
**3c (T)**	0	0	1
**3d**	0.052	0.052	0.967
**3e**	0.018	0.018	1
**4a**	**2.322**	1	0.997
**4b**	0.758	0.758	0.999
**4c (T)**	0	0	1
**4d**	0.399	0.399	0.996
**4e**	0	0	1
**5a**	0.391	0.391	0.995
**5b**	0.123	0.123	0.993
**5c**	0.541	0.541	0.994
**5d**	0	0	1
**5e**	0.697	0.697	0.951
**6a**	0	0	1
**7a**	0.176	0.176	0.998
**7b**	0	0	1
**8a**	0	0	1
**8b**	0	0	1
**8c**	0.004	0.004	0.993
**9a**	0	0	1
**9b (T)**	0	0	1
**9c**	**6.772**	1	0.995
**10a**	0.211	0.211	1
**10b**	0.084	0.084	0.980
**10c**	0.027	0.027	0.985
